# Unaltered Striatal Dopamine Release Levels in Young *Parkin* Knockout, *Pink1* Knockout, *DJ-1* Knockout and LRRK2 R1441G Transgenic Mice

**DOI:** 10.1371/journal.pone.0094826

**Published:** 2014-04-14

**Authors:** Gonzalo Sanchez, Rafael K. Varaschin, Hansruedi Büeler, Paul C. Marcogliese, David S. Park, Louis-Eric Trudeau

**Affiliations:** 1 Departments of pharmacology and neurosciences, Central Nervous System Research Group, Faculty of Medicine, Université de Montréal, Montreal, Canada; 2 School of Life Sciences and Technology, Harbin Institute of Technology, Harbin, China; 3 Department of cellular and molecular medicine, Faculty of Medicine, University of Ottawa, Ottawa, Canada; National Institutes of Health, United States of America

## Abstract

Parkinson's disease (PD) is one of the most prevalent neurodegenerative brain diseases; it is accompanied by extensive loss of dopamine (DA) neurons of the substantia nigra that project to the putamen, leading to impaired motor functions. Several genes have been associated with hereditary forms of the disease and transgenic mice have been developed by a number of groups to produce animal models of PD and to explore the basic functions of these genes. Surprisingly, most of the various mouse lines generated such as *Parkin* KO, *Pink1* KO, *DJ-1* KO and *LRRK2* transgenic have been reported to lack degeneration of nigral DA neuron, one of the hallmarks of PD. However, modest impairments of motor behavior have been reported, suggesting the possibility that the models recapitulate at least some of the early stages of PD, including early dysfunction of DA axon terminals. To further evaluate this possibility, here we provide for the first time a systematic comparison of DA release in four different mouse lines, examined at a young age range, prior to potential age-dependent compensations. Using fast scan cyclic voltammetry in striatal sections prepared from young, 6–8 weeks old mice, we examined sub-second DA overflow evoked by single pulses and action potential trains. Unexpectedly, none of the models displayed any dysfunction of DA overflow or reuptake. These results, compatible with the lack of DA neuron loss in these models, suggest that molecular dysfunctions caused by the absence or mutation of these individual genes are not sufficient to perturb the function and survival of mouse DA neurons.

## Introduction

The main symptoms of Parkinson's disease (PD) including tremor, rigidity and slowness of movements are due in part to the degeneration of dopamine (DA) neurons of the substantia nigra *pars compacta* (SNc) and the associated loss of dopaminergic inputs to the striatum, leading to perturbation of basal ganglia circuits [1]. Although multiple populations of neurons appear to be perturbed at different stages of the disease, DA neurons of the SNc are thought to be particularly vulnerable to environmental and genetic insults, which are believed to interact to cause PD [2]. Both sporadic and familial forms of PD are age-related and thought to implicate cellular dysfunctions including perturbed mitochondrial quality control, impaired reactive oxygen species scavenging and protein aggregation, leading to impaired axonal function and DA release, eventually leading to cell death [3]–[8].

Several transgenic mouse lines have been generated to study the role of PD-related genes in motor behavior, neuronal survival and function and DA release. Knock out approaches have been widely used for autosomal recessive alleles including *parkin*, *DJ-1* and *Pink1* [9]–[15], while knockin, knockout and BAC driven overexpression strategies have been used to evaluate the implication of potential gain of function mutant alleles of genes such as *LRRK2* [16], *parkin* [17] or alpha-synuclein [18], [19]. A number of molecular perturbations have been shown to result from these gene defects including fragmented and dysfunctional mitochondria [6], [7], [15], [20]–[22], altered mitophagy [23]–[27] and altered reactive oxygen species and calcium handling [28]–[30]. However, studies evaluating the structural integrity of the nigrostriatal DA system in most of these genetically-modified mice failed to identify significant loss of DA neurons or DAergic markers [9], [11], [16], [18], [31]–[34], although increased sensitivity to the neurotoxic effects of MPTP have been reported [10], [35]–[37]. Finally, a number of authors reported mild alterations in locomotor function in Parkin KO, DJ-1 KO, Pink1 KO and LRRK2 transgenic mice [9], [31], [33], [34], [38]. Interestingly, a recently described DJ-1 KO mouse backcrossed on a C57/BL6 background showed unilateral loss of DA neurons at 2 months in a subset of animals, suggesting that the penetrance of some of the gene defects examined previously can vary depending on some unknown modifying genes [39]. Although no major loss of DA neurons occurs in most PD genetic models, if mitochondrial dysfunctions also occur in DA neurons in these mice, this could perturb ATP supply and calcium handling in axon terminals and lead to perturbed DA release, especially since DAergic axon terminals are suspected of having particularly high energy requirements [40] and because of the purported heightened vulnerability of axonal mitochondria [41]. A similar hypothesis has been raised in Alzheimers' disease mouse models, in which synaptic dysfunction in hippocampus has been shown to precede plaque formation and cell loss by many months in some models [42]. A number of studies have thus been performed to study DA release in mouse genetic PD models using electrochemical techniques such as amperometry, fast scan cyclic voltammetry (FSCV) and microdialysis coupled to HPLC. Although only a limited number of studies have been performed, most of them using amperometry, these studies have provided support for the existence of mild impairments in activity-dependent DA overflow in the striatum, attributed either to impaired DA release or increased reuptake: while decreased DA overflow has been reported to occur in Parkin KO mice [12], [14], PINK1 KO mice [11] and LRRK2-G2019S [16] or LRRK2-R1441G [34] transgenic mice, evidence for increased DA reuptake has been reported in DJ-1 KO mice [33]. Of note, in most of these studies DA release was triggered by single pulses (or spontaneous firing) and only one has evaluated DA release in response to more energetically-demanding pulse trains [14] (but see Platt et al., for a recent evaluation of DA overflow by pulse trains in A53T alpha-synuclein transgenic mice [43]. Furthermore, in most of these studies, mice were examined at ages of 3 months and older. In this context, it is interesting to note that Oyama and colleagues reported reduced in vivo DA overflow evoked by 50 Hz stimulation trains in 3 and 6 months old male Parkin KO mice, but not in 9 or 12 months old mice [14], arguing for the existence of age-dependent compensations. It would thus be important to conduct a comparative reevaluation of activity-dependent DA overflow in multiple genetic models at ages below 3 months.

In the present study, we used FSCV to provide a comparative evaluation of DA overflow evoked by single pulses, paired pulses and pulse trains in striatal slices prepared from 6–8 weeks-old male transgenic mice, comparing Parkin KO, DJ-1 KO, Pink1 KO and LRRK2 R1441G transgenic mice. Our results fail to detect any significant changes in DA overflow parameters in these mice.

## Materials and Methods

### Animals

All procedures were approved by the animal ethics committee (CDEA) of the Université de Montréal (protocol number 12–161). All efforts were made to minimize animal suffering. Parkin KO mice (WT n = 11; KO n = 14) were a kind gift of Dr. Alexis Brice [44]. DJ-1 KO mice (WT n = 9; KO n = 10) were a kind gift of Dr. Tak Mak [10]. Pink1 KO mice (WT n = 6; KO n = 6) were described in our previous work [15]. LRRK2 (R1441G) transgenic mice (WT n = 10; KO n = 10) were purchased from The Jackson Laboratory (stock # 009604) [34]. Animals were housed with water and food *ad libitum* and a light cycle of 12/12 (lights on at 6:00 AM).

### Brain slice preparation and solutions

Six to eight weeks old male mice were anesthetized with halothane and promptly decapitated. Their brain was quickly removed after gentle opening of the skull and placed in ice-cold artificial cerebrospinal fluid (ACSF) (in mM): 125 NaCl, 26 NaHCO_3_, 2.5 KCl, 2.4 CaCl_2_, 1.3 MgSO_4_, 0.3 KH_2_PO_4_ and 10 D-Glucose; adjusted to 300 mOsm/kg and saturated with 95% O_2_–5% CO_2_). 300 µm thick coronal brain slices containing the most rostral portion of the striatum (as defined by Bregma coordinates: from 1.42 to 0.14 mm [45] were prepared with a VT1000S vibratome (Leica Microsystems Inc., Nussloch, Germany) in ice-cold ACSF, and then kept in ACSF at room temperature in a custom-made submerged recovery chamber for at least 1 hour. Slices were transferred to a custom-made submerged recording chamber superfused with ACSF (approximately 1 ml/min, gravity driven) and maintained at 32°C with a TC-324B single channel heater controller (Warner Instrument Inc., Hamden, CT, USA). WT and transgenic littermates were alternatively euthanized in each experiment to account for the different time elapsed between slice preparation and FSCV recordings. The number of experimental observations (“n”) refers to the number of slices. One or two slices per animal were used.

All drugs and chemicals were obtained from Sigma-Aldrich Canada (Oakville, ON). A stock solution of nomifensine was prepared in H_2_O (3 mM) and stored at 4°C; it was directly diluted into circulating ACSF during the experiment and 5 minutes later electrical stimulation resumed.

### Electrochemical recordings

Action potential-induced DA overflow transients were evoked by electrical stimulation with a bipolar electrode (Plastics One, Roanoke, VA) and detected by FSCV using a carbon-fiber electrode placed into the dorsal striatum, approximately 100 µm below the surface. Carbon-fiber electrodes were fabricated as previously described [46]. Briefly, carbon fibers (Cytec Industries Inc., NJ, USA) approximately 5 µm in diameter were aspirated into ethanol-cleaned glass capillaries (1.2 mm 60.68 mm, 4 inches long; A-M Systems, WA, USA). The glass capillaries were then shaped using a P-2000 micropipette puller (Sutter Instruments, Novato, USA), dipped into 90°C epoxy for 30 s (Epo-Tek 301, Epoxy Technology, MASS, USA) and cleaned in hot acetone for 3 s. The electrodes were heated at 100°C for 12 h and 150°C for 5 days. Before and after usage, electrodes were cleaned with isopropyl alcohol to promote greater sensitivity. Carbon fibers were cut using a scalpel blade under direct visualization to obtain maximal basal currents in ACSF between 100 and 180 nA. Electrodes were finally selected for their sensitivity to DA using in vitro calibration with 1 µM DA in ACSF. The potential of the carbon fiber electrode was scanned at a rate of 300 V/s according to a 10 ms triangular voltage waveform (−400 to 1000 mV vs Ag/AgCl reference) with a 100 ms sampling interval using an Axopatch 200B amplifier (Molecular Devices, Sunnyvale, CA). Data were acquired using a DigiData 1440A analog to digital board converter (Molecular Devices) connected to a computer running Clampex 10 (Molecular Devices). Stimulation (1 ms long monophasic pulses of 400 µA) was generated by a S-900 stimulator (Dagan Corporation, Minneapolis, MN) every 2 min to evoke DA release (single pulse, 100 Hz double pulse or 10 Hz 20 pulses train). FSCV was done with the same electrode for at least one pair of slices corresponding to each genotype during the same day of experiment. Recordings in which peak DA overflow showed run-down greater than 20% between the first and the fifth response to single stimulation were considered unstable and not used for further analyses and a new slice was tested. Rejection frequencies did not differ between WT and Tg groups in any of the 4 different colonies (Parkin WT 2 out of 13 and KO 4 out of 22; DJ-1 all slices reached criterion; Pink1 WT 1 out of 13; LRRK2 WT 2 out of 13 and OE 1 out of 14).

To estimate the rate of DA re-uptake, a single exponential model was fitted to the single pulse evoked DA transients from the peak to the end of the signal [47]; the down stroke of the transient is known to represent the clearance of DA by concurrent reuptake and diffusion. The model had a good fit to the data (goodness of fit medians with 25 and 75% percentiles: 0.9726 (0.8230–0.9867).

### Statistical analysis

Statistical comparisons were carried out with Prism 5 (GraphPad software); significance level was set at 0.05. All data samples were tested for normal distribution to choose between parametric versus non parametric statistics and to represent population parameters either as mean ± standard error (SEM) or as median with 25% percentile range; figure legends state the specific test employed in each case.

## Results

FSCV was used to record electrically-evoked DA overflow in the dorso-lateral aspect of the striatum in brain slices prepared from 6–8 weeks old KO and matched WT littermate animals. Single pulses, double pulses (two pulses separated by a 10 ms interval) and train stimulation (20 pulses at 10 Hz) were used to trigger DA overflow under conditions of increasing energy demands. DA overflow was also evoked in the same slices under conditions of DA transporter (DAT) blockade (5 µM nomifensine) so as to evaluate changes in DA release that may be masked by DA reuptake and to detect possible changes in DAT function.

**Figure 1:**
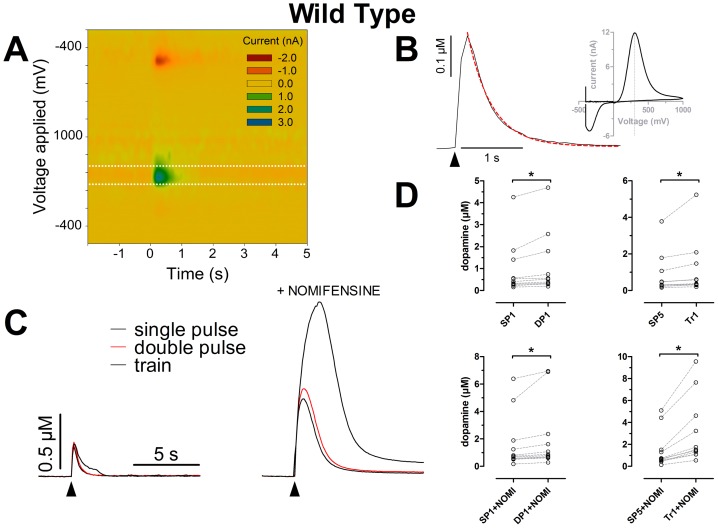
Dopaminergic transmission evaluated using FSCV. Electrically evoked DA transients were detected by FSCV in the dorso-lateral region of slices containing the most rostral part of the striatum. **A** Heat-map plot showing the measured current as a function of time and applied potential. **B** Extracellular DA overflow measured by the carbon fiber electrode at a sampling rate of 10 Hz (black trace) and the exponential fit (red trace) applied from the peak to the end of the signal (half-life: 0.2723 s, 95% confidence intervals: 0.2633 to 0.2821 s; R^2^: 0.9646); arrow-head represents the time of stimulation. Inset: voltammogram corresponding to the peak of DA concentration, depicting maximal oxidation current at a potential of 306.12 mV (vertical dotted line). **C** Averaged 5 consecutive responses to either single-pulse, double pulse or train stimulation in normal ACSF (left) or in the presence of 5 µM nomifensine. **D** Scatter plots representing the peak DA concentration elicited by the different stimuli. Significant differences were found between the first single and double pulse stimulation in normal ACSF and between the fifth single pulse and the first train (p = 0.0010; p = 0.0020, respectively. Two-tailed Wilcoxon matched pairs test). In the presence of 5 µM nomifensine both comparisons yielded significant differences (both p = 0.0010. Two-tailed Wilcoxon matched pairs-test). All data presented in this figure were generated from experiments with WT animals belonging to the Parkin colony (slices n = 11; animals n = 11).

6–8 week old mice carrying a deletion of the *parkin* gene were first examined [44]. Single pulses and double pulses were first administered in an alternating fashion every 2 min for a total period of 20 min. After 20 min, train stimulation was administered for an additional 10 min period. All slices were subsequently exposed to nomifensine (5 µM) in the presence of which the complete protocol was carried out a second time. In order to validate our experimental design we first compared maximal DA overflow produced in striatal slice from WT mice by the different stimuli employed. The heat-map in figure 1A depicts the color coded voltammetric current as a function of applied potential and time. Figure 1B shows a DA transient evoked by single pulse stimulation and the fit of the exponential model to estimate DA clearance. A background-subtracted cyclic voltammogram confirms that the detected analyte is DA.

The concentration of DA at the peak of the transients elicited by single pulse stimulation was statistically different from that evoked by double pulses (Fig. 1C). The first single pulse stimulation in WT animals evoked an average DA peak of 0.41±0.28 µM (median ±25% percentile range) (Fig. 1D, left upper panel). The first double pulses stimulation evoked a DA peak of 0.52±0.30 µM (median ±25% percentile range), which was significantly increased in comparison to the single pulse response (n = 11 two tailed Wilcoxon matched pairs test p = 0.0010). Comparing the last single pulse (0.47±0.28 µM, median ±25% percentile range) to the first train stimulation (0.58±0.31 µM, median ±25% percentile range), also yielded a significant difference in peak amplitude (p = 0.0020 n = 11 two-tailed Wilcoxon matched pairs test. Fig 1D, right upper panel).

In the presence of the DA transporter blocker nomifensine, the first single pulse response (0.73±0.54 µM, median ±25% percentile range) was significantly different from the first double pulse response (0.87±0.28 µM, median ±25% percentile range) (two-tailed Wilcoxon matched pairs test p = 0.0010; n = 11. Fig 1D, left lower panel). Likewise, the last single pulse response (0.69±0.48 µM) was statistically different from the first train response (1.51±1.09 µM) (medians ±25% percentile range; two-tailed Wilcoxon matched pairs test p = 0.0010; n = 11. Fig 1D, right lower panel p = 0.0010; n = 11), indicating that the different stimuli were mobilizing different amounts of neurotransmitter vesicles from the releasable pool in axon terminals.

**Figure 2:**
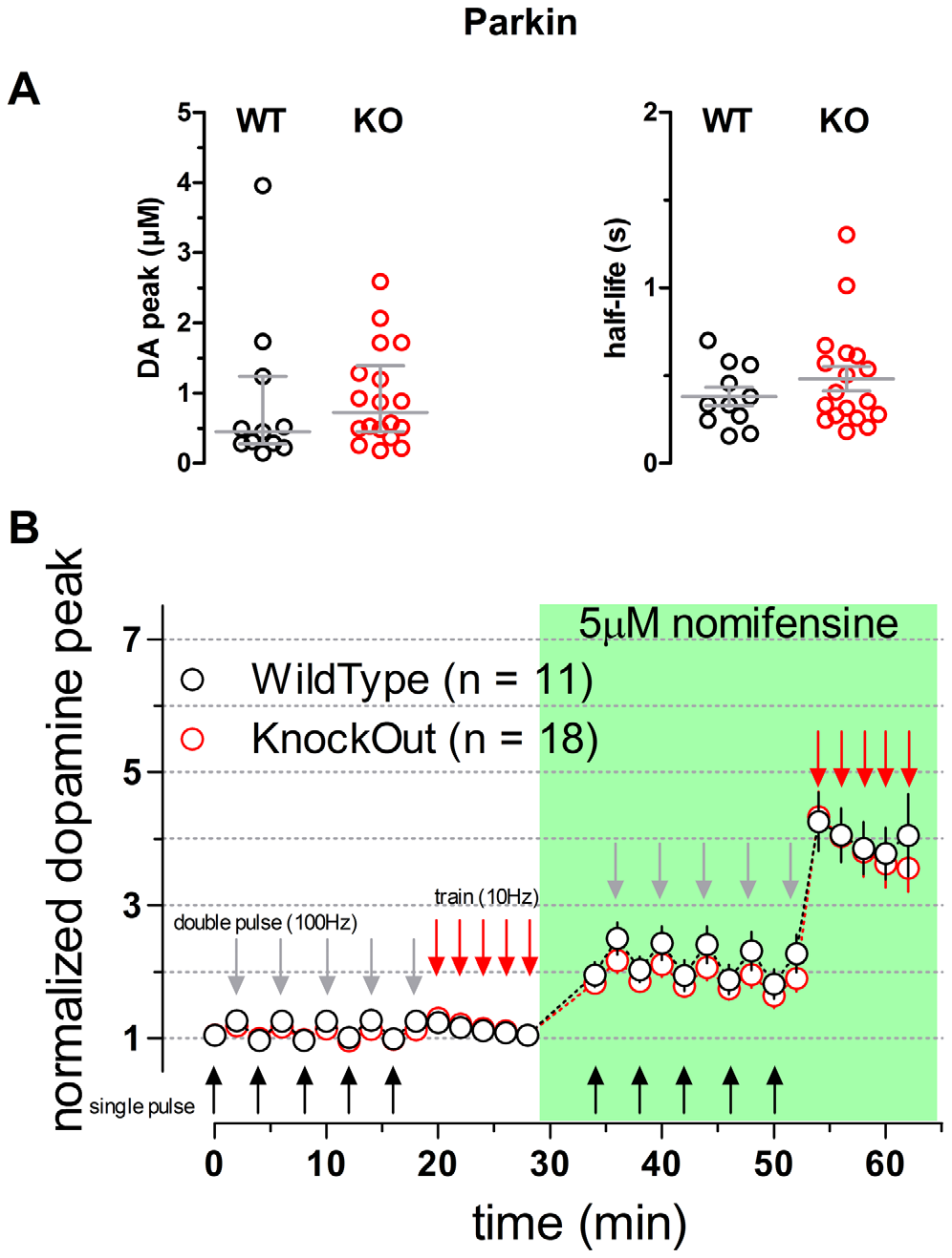
Dopaminergic transmission in striatal slices of parkin WT and KO mice. **A** Scatter plots illustrating the values of the peak [DA] (left panel) and the half-life (right panel) measured from averaged single-pulse transients. Bars represent median and 25% interquartile range (DA peak) and mean and SEM (half-life), no statistical differences were detected when comparing the WT and KO genotypes (Two-tailed Mann Whitney p = 0.2909 and two-tailed t test p = 0.3109 DA peak and half-life, respectively). **B** Time course of the normalized peak of the evoked responses to single pulses (black arrows) double pulses (gray arrows) and trains (red arrows). Genotypes were compared by means of a 2 Way Repeated Measures ANOVA yielding no significant difference (p = 0.5478; slices n = 11, animals n = 11 WT; slices n = 18, animals n = 14 KO). Green box indicates presence of 5 µM nomifensine into circulating ACSF.

Comparing slices obtained from *parkin* KO and WT littermate controls, a two way repeated measures ANOVA comparing the peaks of DA transients normalized to the first evoked response provided no evidence for any difference between genotypes (n = 11 WT and n = 18 KO; p = 0.5478) (Fig. 2B). An analysis of non-normalized peak DA levels detected in response to single pulses confirmed a lack of difference between genotypes (0.45±0.28 µM n = 11 WT, 0.72±0.45 µM n = 18 KO medians ±25% percentile range; p = 0.2909 two-tailed Mann Whitney test; Fig. 2A, left), compatible with the absence of any impairment in activity-dependent DA release. The half-life of DA overflow responses evoked by single pulses was also not different between genotypes (0.38±0.05 s for WT and 0.48±0.07 s for KO, means ± SEM; student t test P value  = 0.3109) (Fig. 2A, right), suggesting that DAT function was not altered.

**Figure 3:**
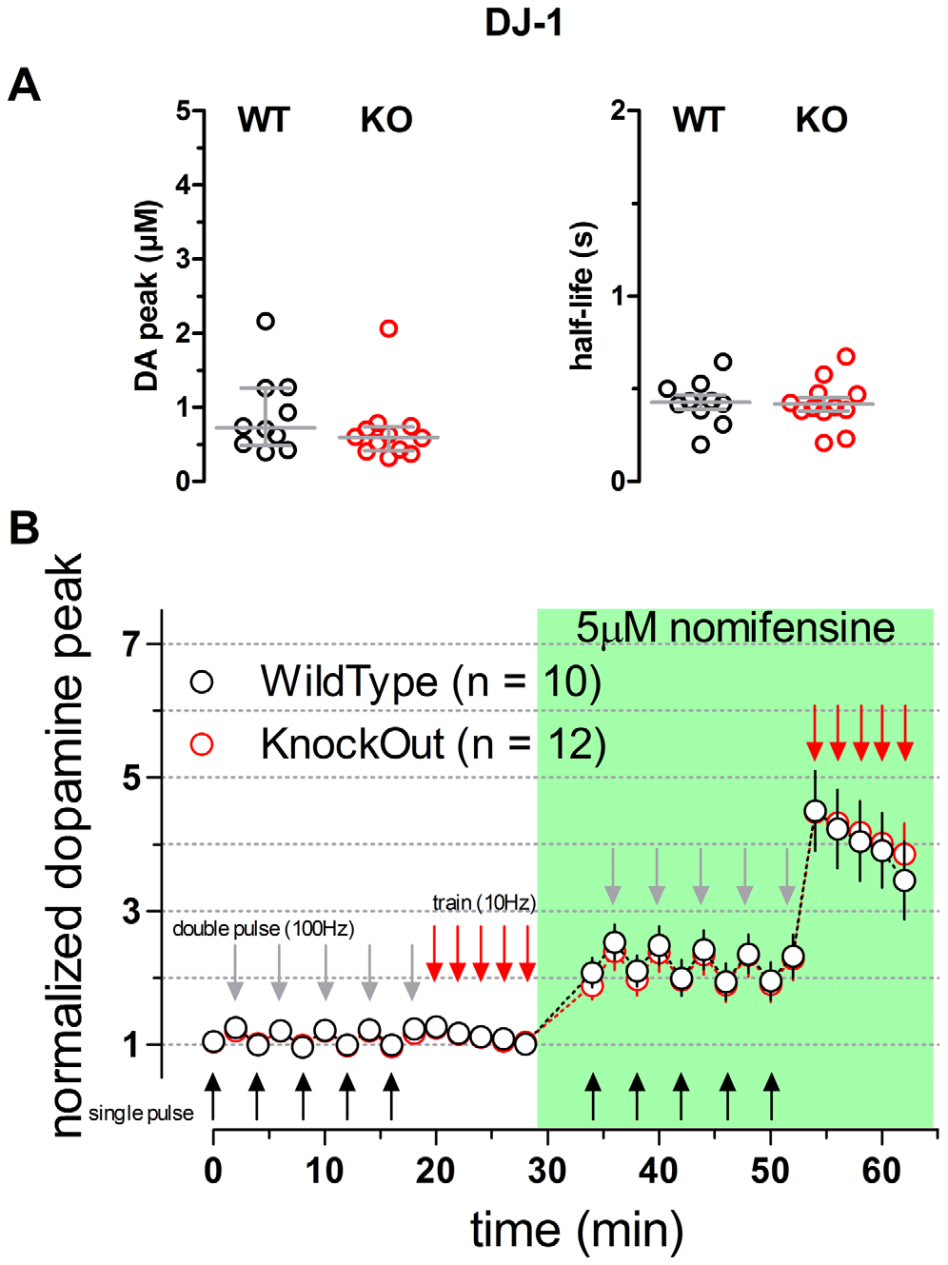
Dopaminergic transmission in striatal slices of DJ-1 WT and KO mice. **A** Scatter plots illustrating the values of the peak [DA] (left panel) and the half-life (right panel) measured from averaged single-pulse transients. Bars represent median and 25% interquartile range (DA peak) and mean and SEM (half-life), no statistical differences were detected when comparing the WT and KO genotypes (Two-tailed Mann Whitney p = 0.2225 & two-tailed t test p = 0.8562 DA peak and half-life, respectively). **B** Time course of the normalized peak of the evoked responses to single pulses (black arrows) double pulses (gray arrows) and trains (red arrows). Genotypes were compared by means of a 2 Way Repeated Measures ANOVA yielding no significant difference (p = 0.9679; slices n = 10, animals n = 9 WT; slices n = 12, animals n = 10 KO). Green box indicates presence of 5 µM nomifensine into circulating ACSF.

6–8 week old mice carrying a deletion of the DJ-1 gene (Kim et al., 2005) were next examined using the same stimulation protocol (Fig. 3). A two way repeated measures ANOVA comparing WT and KO data sets collected in the absence or in the presence of nomifensine provided no evidence for any difference between genotypes (n = 10 WT and n = 12 KO; p = 0.9679). An analysis of raw peak DA levels detected in response to single pulses confirmed a lack of difference between genotypes (WT median: 0.72 µM; 25% percentile: 0.49; KO median 0.60 µM; 25% percentile: 0.41; p = 0.2225 Mann Whitney two-tailed test; Fig. 3A, left). The half-life of DA overflow responses evoked by single pulses was also not different between genotypes (WT mean of 0.43±0.04 s; KO 0.42±0.04 s; student t test P value  = 0.8562; Fig. 3A, right).

**Figure 4:**
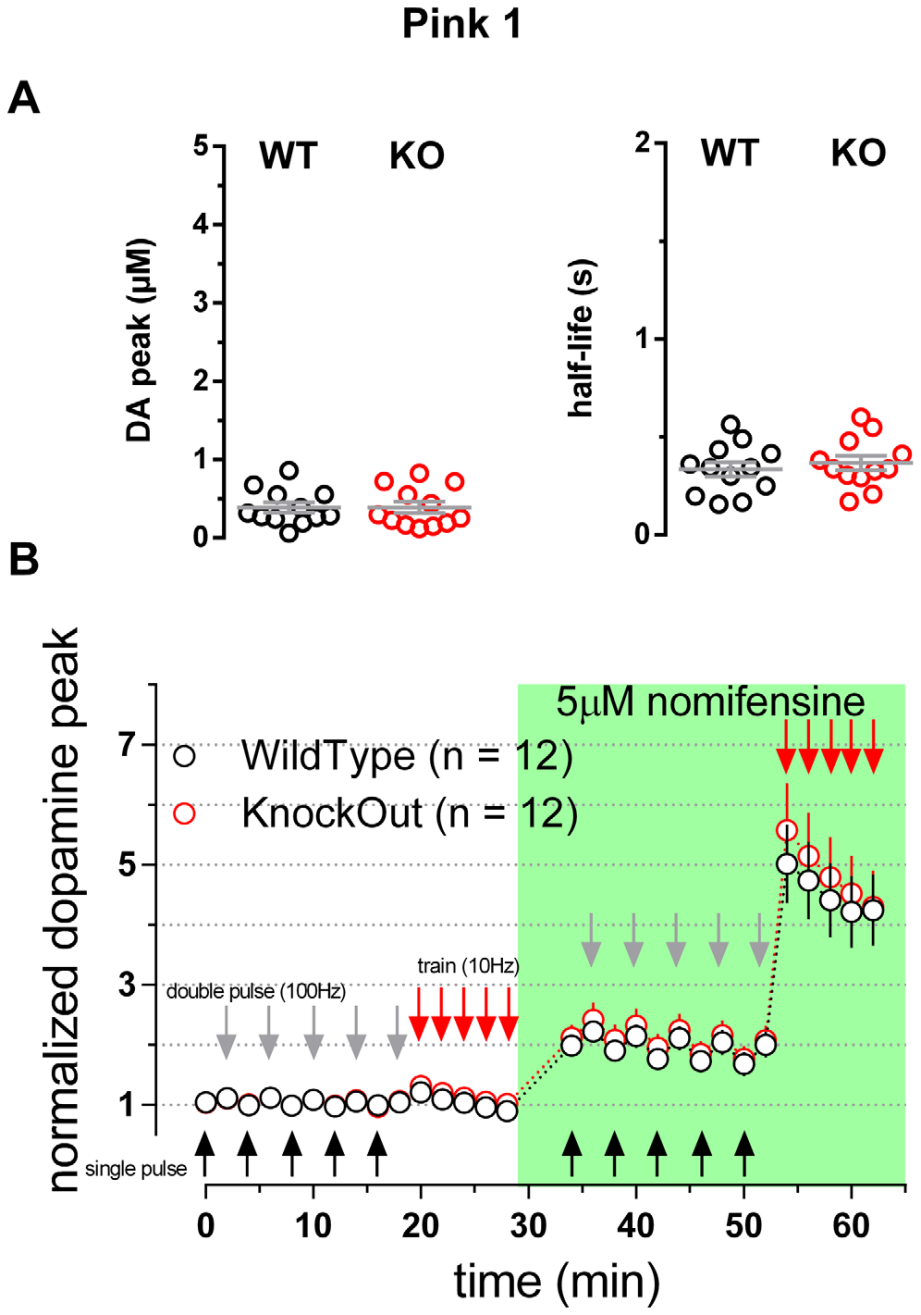
Dopaminergic transmission in striatal slices of Pink1 WT and KO mice. **A** Scatter plots illustrating the values of the peak [DA] (left panel) and the half-life (right panel) measured from averaged single-pulse transients. Bars represent mean and SEM, no statistical differences were detected when comparing the WT and KO genotypes (two-tailed unpaired t test p = 0.995 and p = 0.556 DA peak and half-life, respectively). **B** Time course of the normalized peak of the evoked responses to single pulses (black arrows) double pulses (gray arrows) and trains (red arrows). Genotypes were compared by means of a 2 Way Repeated Measures ANOVA yielding no significant difference (p = 0.2100; n = 12 WT and n = 12 KO). The green box indicates the presence of 5 µM nomifensine into circulating ACSF.

6-8 week old mice carrying a deletion of the Pink1 gene (Akundi et al., 2011) were also examined (Fig. 4). A two way repeated measures ANOVA comparing WT and KO data sets collected in the absence or in the presence of nomifensine provided no evidence for any difference between genotypes (n = 12; p>0.05). An analysis of raw peak DA levels detected in response to single pulses confirmed a lack of difference between genotypes (0.386±0.066 µM and 0.387±0.073 µM, mean ± SEM of WT and KO respectively; n = 12; p>0.05; Fig. 4A, 4B). The half-life of DA overflow responses evoked by single pulses was also not different between genotypes (0.34±0.04 s and 0.37±0.04 s mean ± SEM of WT and KO respectively; n = 12; p>0.05; Fig. 4A).

**Figure 5:**
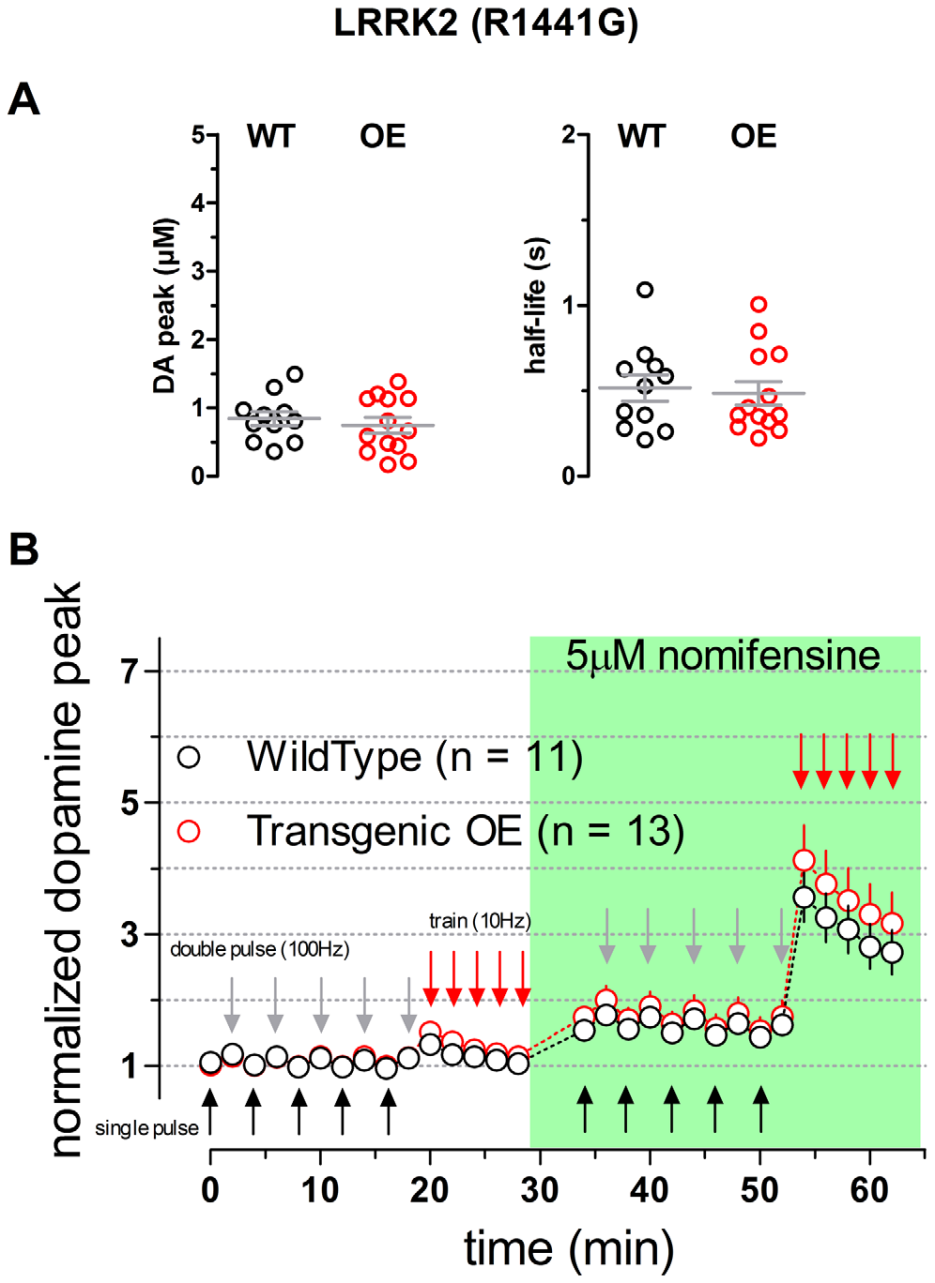
Dopaminergic transmission in striatal slices of WT and LRRK2(R1441G) transgenic mice. **A** Scatter plots illustrating the values of the peak [DA] (left panel) and the half-life (right panel) measured from averaged single-pulse transients. Bars represent mean and SEM, no statistical differences were detected when comparing the WT and transgenic genotypes (two-tailed unpaired t test p  = 0.5421 and p = 0.7671 DA peak and half-life, respectively). **B** Time course of the normalized peak of the evoked responses to single pulses (black arrows) double pulses (gray arrows) and trains (red arrows). Genotypes were compared by means of a 2 Way Repeated Measures ANOVA yielding no significant difference (p = 0.4337; n = 11 WT and n = 13 Tg, animals n = 10 each). The green box indicates the presence of 5 µM nomifensine into circulating ACSF.

Finally, 6–8 week old transgenic mice carrying a mutated version of the LRRK2 gene (R1441G) [34] were examined with the same protocol. A two way repeated measures ANOVA comparing WT and homozygous LRRK2 transgenic data sets collected in the absence or in the presence of nomifensine provided no evidence for any difference between genotypes (p = 0.4337; Fig. 5). An analysis of non-normalized DA levels detected in response to single pulses confirmed a lack of difference between genotypes (WT n = 11: 0.84±0.10 µM, mean ± SEM, Tg n = 13: 0.75±0.11 µM, mean ± SEM; two-tailed student t test p = 0.5421; Fig. 5A, 5B).

The half-life of DA overflow responses evoked by single pulses was also not different between genotypes (0.52±0.08 s, mean ± SEM for WT and 0.49±0.07 s, mean ± SEM for LRRK2 Tg; two-tailed student t test p = 0.7671; Fig. 5A).

## Discussion

This is the first study to provide a side by side comparative evaluation of striatal DA transmission in some of the main KO or transgenic mouse models of PD (parkin, DJ-1, PINK1 and LRRK2-R1441G Tg mice). Considering the previously reported absence of DA neuron loss in these mice and the possibility of age-dependent compensatory adaptations capable of circumventing primary impairments [3], [14], [16], [48], [49], we restricted our analysis to striatal brain slices prepared from young 6–8 week old mice. In light of the multiple studies suggesting mitochondrial dysfunction and related bioenergetic defects due to mutations in these genes [3], we examined activity-dependent DA overflow induced by graded stimuli ranging from single pulses, paired pulses and trains. Using FSCV, a technique allowing direct detection of evoked DA transients with subsecond resolution, we found that paired pulses and stimulation trains induced more extensive DA release, most likely recruiting vesicles from both the readily releasable and reserve pools. We found no evidence of impaired DA axon terminal function in any of the strains tested, thus excluding any robust effect of the genotypes on both DA release and reuptake from nigrostriatal terminals at the age range examined. Subtle impairments of DA transmission cannot be excluded in light of the present results; additional experiments such as input/output analysis, evaluations of the rate of recovery of paired pulse suppression and its dependence on extracellular [Ca^2+^] could provide further insight into other possible alterations of the release machinery in dopaminergic neurons [50]–[52].

Our findings are intriguing in light of the previous reports suggesting either reduced release or enhanced reuptake in older genetically-modified mice. For example, evaluating female Parkin KO mice of 8–16 weeks using amperometry, Kitada and colleagues [12] reported reduced DA overflow in response to single pulses. In a separate study, using a different Parkin KO mouse line, Oyama and colleagues reported reduced *in vivo* DA overflow evoked by 50 Hz stimulation trains in 3 and 6 months old male mice, but not in 9 or 12 months old mice [14]. In DJ-1 KO mice, a single study reported unaltered DA release measured by amperometry and evoked by single pulses in 3 months old mice, but functional evidence for increased DAT function [33]. In 2–3 months old Pink1 KO mice, amperometric recordings suggested reduced DA overflow induced by single pulses [11]. In male LRRK2 G2019S transgenic mice, FSCV was used to show reduced DA overflow evoked by single pulses in 12 months old KO mice but not in 6 months old mice [16]. The reason for our inability to detect similar perturbations of DA axon terminal functions in the present study is unclear. The age of the mice could represent an important variable to consider. In the present study, 6–8 weeks old mice were used, which is younger than most of the previous studies. It is thus possible that deficits only appear at older ages. However, the study of Oyama and colleagues [14] illustrates that early deficits can be compensated at later ages, thus arguing for the importance of evaluating younger mice in order to identify primary, early deficits, that could be relevant to understanding cellular dysfunctions that are related to presymptomatic stages of PD. In any case, our findings highlight the fact that deleting Parkin, Pink1 and DJ-1 genes, or overexpressing LRRK2 R1441G does not cause a robust and primary deficit in DA release or DA reuptake in young mice, suggesting that other cellular stressors related to age, environmental toxin exposure or genetic background may be required to lead to robust perturbations of DA overflow. The previous demonstrations that most DJ-1 and Pink1 KO mice do not show loss of DA neurons even at an advanced age (but see [39]), but show increased loss of DA neurons in response to MPTP is compatible with this interpretation [10], [37]. Finally, our findings are in agreement with previous studies showing an absence of DA neuron loss in each of the mouse lines examined here, as well as in Parkin/Pink1/DJ-1 triple knockout mice [13].
